# Artificial intelligence in lung cancer: current applications, future perspectives, and challenges

**DOI:** 10.3389/fonc.2024.1486310

**Published:** 2024-12-23

**Authors:** Dongdong Huang, Zifang Li, Tao Jiang, Chaojuan Yang, Ning Li

**Affiliations:** ^1^ Department of Respiratory and Critical Care Medicine, Center for Respiratory Medicine, the Fourth Affiliated Hospital of School of Medicine, and International School of Medicine, International Institutes of Medicine, Zhejiang University, Yiwu, China; ^2^ Department of Rehabilitation Medicine, Yiwu Central Hospital, Yiwu, China

**Keywords:** artificial intelligence, lung cancer, precision medicine, early diagnosis, personalized treatment

## Abstract

Artificial intelligence (AI) has significantly impacted various fields, including oncology. This comprehensive review examines the current applications and future prospects of AI in lung cancer research and treatment. We critically analyze the latest AI technologies and their applications across multiple domains, including genomics, transcriptomics, proteomics, metabolomics, immunomics, microbiomics, radiomics, and pathomics in lung cancer research. The review elucidates AI’s transformative role in enhancing early detection, personalizing treatment strategies, and accelerating therapeutic innovations. We explore AI’s impact on precision medicine in lung cancer, encompassing early diagnosis, treatment planning, monitoring, and drug discovery. The potential of AI in analyzing complex datasets, including genetic profiles, imaging data, and clinical records, is discussed, highlighting its capacity to provide more accurate diagnoses and tailored treatment plans. Additionally, we examine AI’s potential in predicting patient responses to immunotherapy and forecasting survival rates, particularly in non-small cell lung cancer (NSCLC). The review addresses technical challenges facing AI implementation in lung cancer care, including data quality and quantity issues, model interpretability, and ethical considerations, while discussing potential solutions and emphasizing the importance of rigorous validation. By providing a comprehensive analysis for researchers and clinicians, this review underscores AI’s indispensable role in combating lung cancer and its potential to usher in a new era of medical breakthroughs, ultimately aiming to improve patient outcomes and quality of life.

## 1 Introduction

Lung cancer is a major challenge in oncology, with a substantial impact on global health metrics. Non-small cell lung cancer (NSCLC) is the predominant form, constituting the majority of cases (1, 2). When diagnosed at an advanced stage, the prognosis is quite poor, with long-term survival rates being relatively low (3, 4). However, if detected at an early stage, the chances of survival improve significantly. For those with locally advanced disease, the survival rates are moderate but still concerning (5). Small cell lung cancer (SCLC) is less common but has an even more serious outlook, with survival rates dropping significantly when the disease has spread extensively. The financial and emotional impact of these cancers on patients and their families is considerable, affecting the quality of life in a meaningful way.

The critical importance of early screening for lung cancer has been increasingly recognized, as it substantially enhances the chances of early detection and treatment. However, even those diagnosed at an early stage are not exempt from the risk of relapse (6). Recurrence often leads to the progression to advanced stages, drastically diminishing the survival prognosis. The pathogenesis and progression of lung cancer are governed by intricate regulatory networks, underscoring the complexity of this disease. Furthermore, the precise mechanisms driving lung cancer initiation and progression, chemotherapeutic resistance, and resistance to targeted therapies such as those against Epidermal Growth Factor Receptor (EGFR) and Anaplastic Lymphoma Kinase (ALK), as well as immune resistance, remain areas necessitating further investigation.

A comprehensive glossary of essential artificial intelligence (AI) terminology used in this manuscript is presented in **Table 1**.

**Table 1:** Glossary of key terms artificial intelligence.

Artificial Intelligence (AI)	The ability of computer systems to perform tasks that typically require human intelligence, such as visual perception, speech recognition, decision-making, and language translation.
Machine learning (ML)	A subset of AI that focuses on the development of algorithms and statistical models that enable computer systems to improve their performance on a specific task through experience, without being explicitly programmed.
Deep Learning (ML)	A subset of machine learning based on artificial neural networks with multiple layers, capable of learning complex patterns in large amounts of data.
Natural Language Processing (NLP)	A field of AI that focuses on the interaction between computers and humans using natural language, enabling machines to understand, interpret, and generate human language.
Computer vision	A field of AI that enables computers to gain high-level understanding from digital images or videos, aiming to automate tasks that the human visual system can do.
Robotics	The branch of technology that deals with the design, construction, operation, and use of robots, often incorporating AI for decision-making and task execution.
Deep neural networks (DNNs)	Artificial neural networks with multiple layers between the input and output layers, capable of modeling complex non-linear relationships.
Convolutional Neural Networks (CNNs)	A class of deep neural networks most commonly applied to analyze visual imagery, designed to automatically and adaptively learn spatial hierarchies of features.
Recurrent Neural Networks (RNNs)	A class of neural networks where connections between nodes form a directed graph along a temporal sequence, allowing it to exhibit temporal dynamic behavior.
Support vector machines (SVMs)	Supervised learning models used for classification and regression analysis, effective in high-dimensional spaces.
Principal Component Analysis (PCA)	A statistical procedure that uses orthogonal transformation to convert a set of observations of possibly correlated variables into a set of values of linearly uncorrelated variables.
t-Distributed Stochastic Neighbor Embedding (t-SNE)	A machine learning algorithm for visualization that reduces dimensionality based on similarity of datapoints.
Uniform Manifold Approximation and Projection (UMAP)	A dimension reduction technique that can be used for visualization similarly to t-SNE, but also for general non-linear dimension reduction.
Principal Component Analysis (PCA)	AI systems that can provide human-understandable explanations for their decisions or predictions.
Explainable Artificial Intelligence (XAI)	AI systems that can provide human-understandable explanations for their decisions or predictions.
Gradient-weighted Class Activation Mapping (Grad-CAM)	A technique for producing visual explanations for decisions made by convolutional neural networks.
Local Interpretable Model-agnostic Explanations (LIME)	A technique that explains the predictions of any classifier in an interpretable and faithful manner.
Area Under the Curve of Receiver Operating Characteristic (AUC-ROC)	A performance measurement for classification problems at various thresholds settings, representing the degree of separability between classes.
Precision-recall curves	A graphical plot that illustrates the trade-off between precision and recall for different thresholds in a binary classifier system.
F1 scores	The harmonic mean of precision and recall, providing a single score that balances both metrics.
Calibration plots	Graphical representations of the agreement between predicted probabilities and observed frequencies, used to assess the calibration of probabilistic predictions.
Decision curve analysis	A method for evaluating and comparing prediction models that accounts for the clinical consequences of using a model.
SHapley Additive exPlanations (SHAP)	A game theoretic approach to explain the output of any machine learning model. It connects optimal credit allocation with local explanations using the classic Shapley values from game theory and their related extensions.

In recent years, AI has emerged as a transformative force in biomedical research, particularly in the domain of lung cancer. AI enhances the potential for early diagnosis through high-precision imaging and pattern recognition, offering new avenues for predicting and monitoring disease progression (7–9). Additionally, AI-driven approaches are crucial in unraveling the complex molecular and genetic landscapes of lung cancer, thus aiding in the identification of novel therapeutic targets and the development of personalized treatment strategies (10, 11). As lung cancer research continues to evolve, the integration of AI technology promises to revolutionize the field, paving the way for more effective and tailored interventions.

AI, a branch of computer science, focuses on creating systems capable of performing tasks traditionally requiring human intelligence. The overarching goal of AI is to enable machines to emulate and execute functions such as perception, learning, reasoning, planning, and natural language processing. Categorically, AI encompasses various technologies including machine learning (ML), deep learning (DL), natural language processing (NLP), computer vision, and robotics (12–14). ML, a pivotal subset of AI, involves techniques that allow computers to learn from data and enhance their performance over time (15). Within ML, methods such as supervised learning, unsupervised learning, semi-supervised learning, and reinforcement learning are employed to extract patterns and make predictions. DL, a further specialized subset of ML, leverages neural networks with multiple layers (deep neural networks, DNNs) to mimic the human brain’s processing mechanism, significantly advancing applications like lung Computed Tomography (CT) radiomics (16). NLP technology enables machines to recognize, understand, and generate human languages, facilitating tasks such as text processing, language translation, semantic analysis, and the development of chatbots (17). In the domain of computer vision, AI grants computers the ability to interpret and understand visual information, encompassing object detection, image classification, and facial recognition, which are particularly relevant in the pathological analysis of lung cancer (18). Robotics, another facet of AI, involves designing and building robots capable of performing tasks requiring human-like intelligence, including perception, movement, and navigation—for instance, the robotic-assisted lung lobectomy (Robot-L) (19). Presently, the applications of AI in the domain of lung cancer research are extensive, encompassing the comprehensive analysis of genomic data, which includes the fields of genomics, transcriptomics, and epigenomics. Additionally, AI methodologies are applied in the realms of radiomics for imaging analysis, digital pathology for the examination of tissue specimens, and the integration and interpretation of real-world multimodal datasets. A notable example is the recent publication in Nature by Harvard University and Massachusetts Institute of Technology, describing PathChat, a versatile AI framework for visual and linguistic analysis in human pathology (20). Through these varied applications, AI not only enhances diagnostic accuracy and therapeutic planning in lung cancer but also promises to usher in a new era of personalized medicine, ultimately improving patient outcomes.

**Figure 1:**
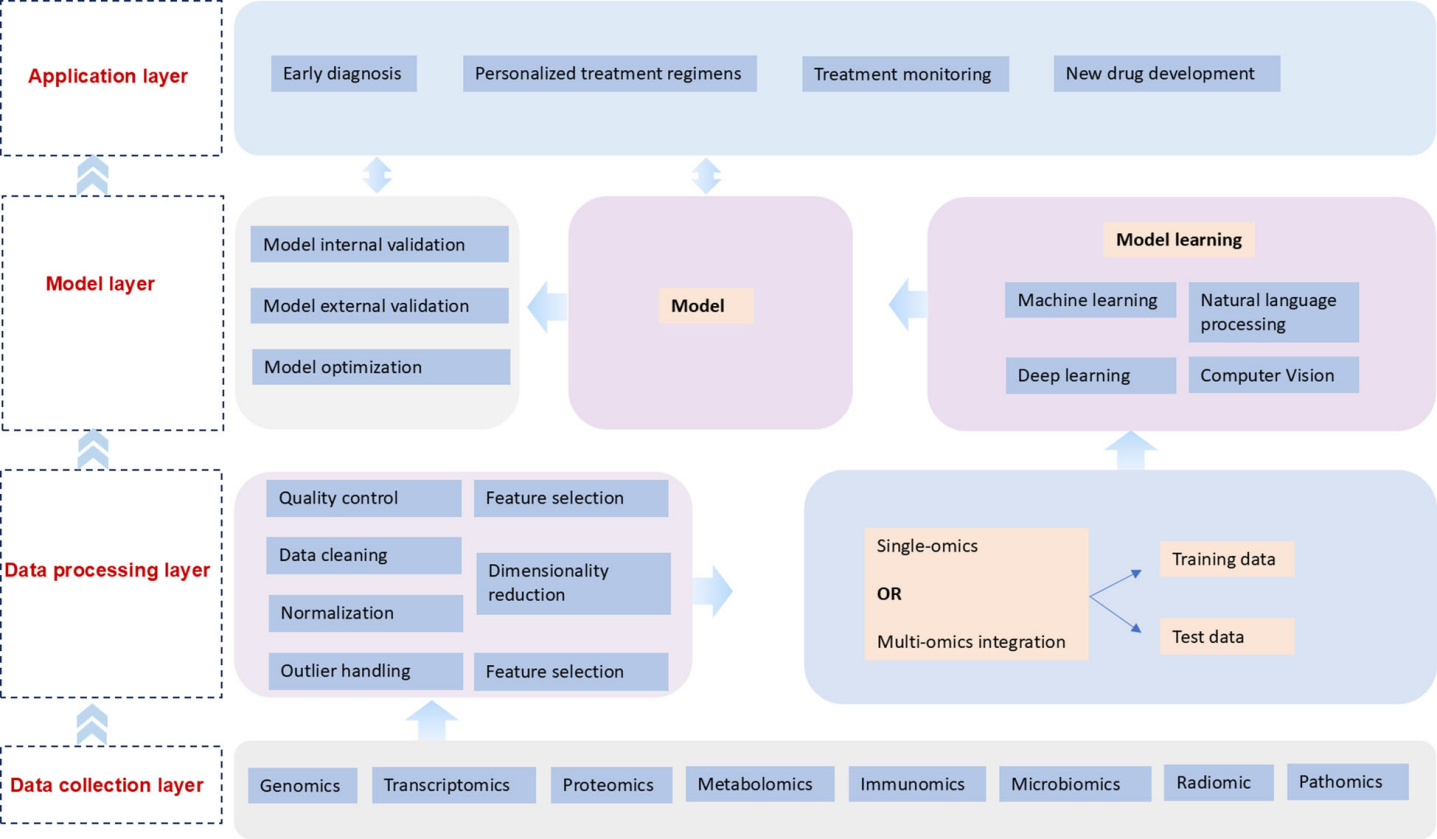
Artificial intelligence-driven multi-omics framework for lung cancer precision medicine.

AI has become a pivotal force in lung cancer research, revolutionizing the methodologies by which we understand, diagnose, and treat this pervasive disease. AI techniques are being increasingly applied to various -omics domains, including genomics, transcriptomics, proteomics, metabolomics, immunogenomics, microbiomics, radiomics, and pathology. These applications have significantly enhanced our ability to decipher complex biological data, providing unprecedented insights into the molecular mechanisms underlying lung cancer. Moreover, AI’s integration into precision medicine promises transformative advancements in early diagnosis, therapeutic monitoring, and novel drug development. In this review, we delve into the multifaceted roles AI plays across these diverse fields, exploring current applications and envisioning the promising future of AI-driven innovations in lung cancer research and treatment. **Figure 1** illustrates how AI transforms diverse biological data to enhance lung cancer diagnosis, personalize treatment, and accelerate drug development, thereby advancing precision oncology. This comprehensive application underscores AI’s immense potential in addressing the full spectrum of lung cancer management, from early screening to therapeutic monitoring and novel drug discovery. As lung cancer research continues to evolve, the integration of AI technologies promises to revolutionize the field, paving the way for more effective and individualized interventions. In this review, we delve into the multifaceted roles AI plays across these diverse domains, exploring current applications and envisioning the promising future of AI-driven innovations in lung cancer research and treatment. We examine how AI’s capabilities in data analysis, pattern recognition, and predictive modeling are being harnessed to improve early detection rates, optimize treatment strategies, and expedite the development of targeted therapies. Furthermore, we discuss the potential of AI to overcome existing challenges in lung cancer management, such as the complexity of tumor heterogeneity and the need for more precise prognostic tools. By critically assessing the current state of AI applications in lung cancer and projecting future developments, this review aims to provide a comprehensive overview of the transformative impact of AI on the landscape of lung cancer research and clinical practice.

## 2 AI in lung cancer: current applications

The application of AI in lung cancer primarily focuses on several areas: genomics, transcriptomics, proteomics, metabolomics, immunomics, microbiomics, radiomics, and pathomics. A comprehensive comparative analysis of AI applications across these omics fields in lung cancer research is presented in **Table 2**.

**Table 2:** Comparative analysis of AI applications across omics fields in lung cancer research.

Omics Field	AI Techniques	Key Applications	Performance/Strengths	Limitations
Genomics	Deep Learning, Machine Learning	- Gene mutation analysis	- High accuracy in mutation detection	- Requires large datasets
		- Genomic instability assessment	- Improved early diagnosis	- Complexity in interpreting genetic variations
		- Epigenetics study	- Non-invasive screening (liquid biopsy)	
		- Liquid biopsy enhancement		
Transcriptomics	Deep Learning, Natural Language Processing	- Gene expression analysis	- Identification of novel biomarkers	- Challenges in handling noise in expression data
		- RNA sequencing data interpretation	- Insight into gene regulation	- Difficulty in interpreting complex gene interactions
Proteomics	Machine Learning, Deep Neural Networks	- Protein-protein interaction prediction	- High-throughput analysis	- Limited by proteome complexity
		- Biomarker discovery	- Identification of drug targets	- Challenges in data integration
Metabolomics	Machine Learning, Deep Learning	- Metabolic pathway analysis	- Early diagnosis potential	- Metabolite variability challenges
		- Biomarker identification	- Treatment response monitoring	- Need for standardized data collection
Immunomics	Deep Learning, Machine Learning	- Immune escape mechanism study	- Personalized immunotherapy approaches	- Complexity of immune system interactions
		- Neoantigen discovery	- Improved understanding of tumor-immune interactions	- Limited availability of comprehensive immune data
		- Immunotherapy response prediction		
Microbiomics	Machine Learning	- Microbiome profile analysis	- Novel insights into lung cancer etiology	- Challenges in standardizing microbiome data
		- Host-microbiome interaction study	- Potential for microbiome-based therapies	- Complexity in interpreting microbial diversity
Radiomics	Convolutional Neural Networks, Deep Learning	- Image feature extraction	- Non-invasive diagnosis	- Variability in imaging protocols
		- Tumor classification	- High accuracy in image analysis	- Need for large, diverse image datasets
		- Treatment response prediction		
Pathomics	Convolutional Neural Networks, Deep Learning	- Automated tissue analysis	- Improved diagnostic accuracy	- Challenges in standardizing tissue preparation
		- Cancer subtype classification	- Efficient histopathological analysis	- Need for extensive pathologist validation

### 2.1 AI in lung cancer genomics

The application of AI in lung cancer genomics encompasses the analysis of gene mutations and variations, the assessment of genomic instability, the study of epigenetics, and the enhancement of non-invasive liquid biopsy techniques. These advancements facilitate early detection, precision treatment, and a comprehensive understanding of the genetic landscape of lung cancer, with the potential to improve patient outcomes and inform therapeutic strategies.

#### 2.1.1 Gene *mutations and variations*

In the realm of lung cancer genomics, AI has revolutionized the identification and analysis of genetic mutations and variations, playing a pivotal role in advancing precision medicine. The integration of DL models, such as convolutional neural networks (CNNs) and recurrent neural networks (RNNs), facilitates the detailed analysis of large-scale genomic data, enabling the precise identification and classification of specific genetic mutations like EGFR, Kirsten Rat Sarcoma virus (KRAS), and ALK (21–24). Additionally, NLP techniques can autonomously extract mutation-related information from scientific literature and genetic databases, thereby enriching the knowledge base for lung cancer genomics (25–27). ML algorithms further extend the capability of AI by estimating the frequency and distribution of particular gene mutations across diverse populations, thus aiding in the formulation of personalized treatment plans (10, 28).

Moreover, AI excels in the integration of multi-omics data for a comprehensive understanding of the impact of genetic mutations on disease progression and prognosis (29–31). This integration not only enhances molecular characterization but also identifies novel therapeutic targets. In drug discovery, virtual screening powered by AI accelerates the identification of potential drug candidates tailored to specific genetic mutations while predicting their efficacy and side effects (32, 33). Individualized therapeutic approaches are meticulously honed by the AI-driven dissection of each patient’s unique mutational profiles, thereby maximizing the efficacy of treatment outcomes.

Predictive models utilizing ML analyze gene mutation data to forecast disease prognosis, treatment responses, and survival rates, thereby assisting clinicians in decision-making processes (24, 34). Furthermore, the application of DL in pathological image analysis automates the identification of tissue and cellular characteristics correlated with specific gene mutations, increasing diagnostic accuracy and efficiency (35–37). Leading AI-based initiatives, such as those by Foundation Medicine, Tempus, and IBM Watson for Oncology, exemplify the transformative potential of AI in enhancing the precision of lung cancer diagnosis and treatment (38). Additionally, AI-driven large-scale databases like The Cancer Genome Atlas (TCGA) and the Genomic Data Commons (GDC) provide invaluable resources for ongoing research and clinical applications.

#### 2.1.2 Genomic instability

In the domain of AI application on genomic instability in lung cancer, significant advancements have been made across various dimensions, including changes in chromosome number, structural alterations of chromosomes, and gene amplification and deletions (39).

Firstly, alterations in chromosome number, such as triploidy and tetraploidy, are hallmark indicators of genomic instability. AI has been leveraged to study these phenomena through two primary modalities: image analysis and genomic sequencing data analysis. For example, DL techniques using CNNs can now automatically analyze chromosomal smears to identify changes in chromosome number and morphology (40). Concurrently, ML algorithms applied to whole-genome sequencing data can detect aneuploidy states, thereby identifying chromosome number instability (28).

Secondly, AI plays a crucial role in detecting chromosomal structural variations such as deletions, duplications, and inversions. Advanced DL models can analyze high-throughput sequencing data with high precision, accurately pinpointing structural variations on chromosomes. Integration of multi-omics data, including genomics and transcriptomics, further enhances AI’s capability to detect structural changes, utilizing ensemble learning models to combine multiple data sources to improve accuracy and sensitivity (41, 42).

Thirdly, gene amplification and deletions represent another aspect of genomic instability in lung cancer. AI’s involvement in this arena includes the detection of gene amplification through AI analysis of copy number variation (CNV) data. Advanced deep learning models such as CNNs and RNNs have been successfully employed to identify CNVs (43, 44). Similarly, ML algorithms can analyze high-throughput sequencing data to detect gene deletions, and recent developments also include the use of AI models to analyze single-cell sequencing data, revealing cellular heterogeneity and gene loss events with higher resolution.

Notable instances of AI application include Google’s DeepVariant, a deep learning tool for genomic research that accurately detects genomic variants, and CopyNumberNet, a deep learning-based algorithm designed to identify CNVs from whole-genome sequencing data (45). Additionally, databases such as TCGA and Gene Expression Omnibus (GEO) provide extensive genomic and gene expression data, which researchers can analyze using AI techniques to identify genomic instability events in lung cancer.

#### 2.1.3 Epigenetics

The integration of AI into lung cancer epigenetics research has marked a significant advancement in understanding the epigenetic alterations associated with lung cancer, which play crucial roles in tumorigenesis, progression, and treatment responses. Unlike genetic changes, epigenetics focuses on the regulatory mechanisms of gene expression, encompassing DNA methylation, histone modifications, non-coding RNA (ncRNA) regulation, and chromatin remodeling (46–50). The potential of AI in lung cancer epigenetics is becoming increasingly evident, particularly in the analysis of DNA methylation and histone modifications, as well as the regulatory roles of ncRNAs.

In the realm of DNA methylation, AI algorithms, including ML and DL, are employed to analyze extensive methylation datasets, enabling the identification of specific methylation patterns associated with lung cancer (51, 52). These patterns serve as biomarkers for early detection and classification of different lung cancer types, and AI-driven analysis of methylation data is utilized to predict patient prognosis, aiding in the development of personalized treatment plans (53, 54). Histone modifications also benefit from the capabilities of AI; by analyzing acetylation and methylation data, AI can construct detailed histone modification maps to elucidate their distribution and changes in lung cancer (55). Moreover, AI algorithms assist in decoding how these modifications influence gene expression, revealing potential regulatory mechanisms that are critical to the pathogenesis of lung cancer.

The regulatory impacts of ncRNAs, particularly microRNAs (miRNAs) and long non-coding RNAs (lncRNAs), are further elucidated through the analytical capabilities of AI. ML models predict miRNA target genes and assess their roles in lung cancer, identifying new therapeutic targets (29, 56, 57). Similarly, AI predicts the expression patterns and potential functions of lncRNAs, uncovering their regulatory effects in lung cancer and aiding in the discovery of novel therapeutic mechanisms (58). Additionally, integrated multi-omics analysis facilitated by AI merges various epigenetic datasets, offering comprehensive insights into the complex regulatory networks underlying lung cancer and allowing for the creation of personalized treatment models based on epigenetic data and clinical information. A notable study utilizing DL models identified multiple DNA methylation sites related to lung cancer prognosis, while integrated analyses revealed coordinated epigenetic regulation of gene expression in lung cancer (30).

These applications underscore AI’s significant potential in decoding the intricate epigenetic landscapes of lung cancer, providing innovative strategies and tools for future research and therapeutic interventions.

#### 2.1.4 Non-invasive liquid biopsy

The application and research of AI in non-invasive liquid biopsy for lung cancer have seen remarkable advancements in recent years, especially in the detection of circulating tumor DNA (ctDNA) from blood samples. Liquid biopsy is an innovative method for diagnosing and monitoring lung cancer by analyzing cancer-related substances in blood or other bodily fluids. Within this domain, AI has significantly enhanced the early detection, monitoring, and personalized treatment of lung cancer. For early diagnosis, AI algorithms coupled with high-sensitivity techniques like digital Polymerase Chain Reaction (dPCR) and next-generation sequencing (NGS) can accurately identify lung cancer-specific mutations and molecular markers from large datasets, distinguishing early-stage lung cancer patients from healthy individuals (59). In terms of monitoring treatment efficacy, ctDNA levels are dynamically measured throughout the treatment process to immediately assess tumor burden and response to therapy (60, 61). AI models can predict treatment outcomes and potential resistance, providing real-time insights (62, 63). Furthermore, AI-driven analysis of ctDNA enables early detection of relapse post-primary treatment by identifying high-risk patients (64, 65). Personalized treatment is another critical area where AI contributes significantly; analyzing the mutation spectrum in ctDNA provides detailed genomic information aiding the selection of the most suitable targeted and immunotherapies (66). Additionally, AI can optimize treatment regimens by integrating ctDNA data with clinical characteristics and treatment history to enhance therapeutic effectiveness (67, 68). Prognostic evaluation is yet another facet where AI demonstrates its utility; it analyzes ctDNA to predict patient survival and disease progression risk, facilitating informed decision-making for treatment strategies and life planning (61, 69). Development of AI-based intelligent diagnostic systems that combine liquid biopsy results with imaging further enhances diagnostic comprehensiveness and accuracy (66). To validate these applications, multicenter clinical trials are essential, ensuring the accuracy and reliability of AI-integrated liquid biopsy approaches.

### 2.2 AI in lung cancer transcriptomics

Transcriptomics in lung cancer involves investigating the changes in gene expression and transcription levels utilizing high-throughput sequencing technologies such as RNA Sequencing (RNA-Seq). This methodology provides comprehensive insights into the messenger RNA (mRNA) and other transcription products within lung cancer cells and tissues, revealing their gene expression profiles. AI has revolutionized the field of lung cancer genomics, particularly in the study and application of lung cancer transcriptomics. The application of AI in the transcriptomics of lung cancer predominantly focuses on gene expression profiling, analysis of ncRNA, mRNA modification, single-cell transcriptomics, spatiotemporal transcriptomics, and transcription factor regulatory networks.

#### 2.2.1 Gene expression profiling

A gene expression profile specifies the particular pattern of genes that are activated or suppressed in lung cancer cells, compared to normal lung cells. Certain genes may be overexpressed, indicating elevated expression levels, while others may be downregulated, showing decreased expression. These distinct gene expression patterns are crucial in identifying lung cancer types, progression stages, and responses to various treatments.

The role of AI in analyzing lung cancer gene expression profiles is multifaceted and pivotal. Firstly, AI excels in handling and analyzing massive, high-dimensional gene expression datasets, efficiently extracting valuable information from complex data (70). Secondly, through ML algorithms like random forests, support vector machines (SVM), and DL, AI can discern key genetic features associated with lung cancer from vast datasets (71, 72). Thirdly, AI models based on gene expression profiles enhance the accuracy of disease classification and diagnosis by distinguishing between benign and malignant tumors or different lung cancer types (73, 74). Furthermore, AI can predict patient prognosis and treatment efficacy using gene expression data, aiding physicians in formulating personalized treatment plans to improve outcomes (75). Additionally, AI facilitates new drug discovery and targeted therapy development by identifying potential drug targets and biomarkers from gene expression profiles, furthering the advancement of precision medicine (76). Lastly, through pathway analysis, AI can identify biological pathways related to lung cancer, deepening our understanding of cancer mechanisms and guiding effective treatment strategies (77).

In summary, AI in lung cancer gene expression profiling significantly enhances diagnostic and therapeutic precision and supports the progression of personalized medicine.

#### 2.2.2 Analysis of ncRNA

In recent years, significant strides have been made in uncovering the role of ncRNAs in lung cancer, facilitated by advancements in AI. ncRNAs, which include miRNAs, lncRNAs, and circular RNAs (circRNAs), do not translate into proteins but are crucial in regulating gene expression, RNA processing, chromatin structure, and various cellular functions. AI techniques have revolutionized the identification and analysis of these ncRNAs, offering promising avenues for lung cancer research and treatment.

First, AI-assisted miRNA profiling has enabled the identification of novel miRNA biomarkers associated with lung cancer, aiding in gene expression regulation. ML methods applied to miRNA datasets have uncovered potential diagnostic and therapeutic targets (78). Similarly, lncRNAs have been the focus of predictive modeling using sophisticated algorithms such as LDAenDL, which can forecast lung cancer-related lncRNA biomarkers (79). These discoveries are critical for gene regulation and chromatin modification, proposing new therapeutic strategies (80).Furthermore, circRNAs have been studied through ML and AI-based integrative analyses, revealing their potential roles in cancer biomarkers and their importance in gene expression and signaling pathways regulation (81). By leveraging the capabilities of AI in big data analytics and pattern recognition, these studies significantly propel the understanding and application of ncRNAs in lung cancer, offering new horizons for diagnostics and treatment advancements.

#### 2.2.3 AI in mRNA modification for lung cancer

In lung cancer transcriptomics, AI advancements have significantly enhanced the study and application of mRNA modifications, particularly N6-methyladenosine (m6A) (82). As an essential player in gene expression, mRNA undergoes methylation, impacting its stability and translation. ML models like SVM, random forests, and DL networks (CNNs, RNNs) have effectively identified m6A modification sites. These technologies utilize high-throughput data, such as RNA-Seq, to elucidate m6A’s effects on mRNA half-life and degradation. Combining transcriptomic and proteomic data, AI has revealed how m6A influences translation efficiency and mRNA localization. AI analyses have also linked m6A patterns to lung cancer prognosis and treatment responses, facilitating the discovery of biomarkers and personalized therapies. This integration of AI has propelled m6A research, offering promising insights into lung cancer diagnostics and therapeutics.

#### 2.2.4 AI in single-cell transcriptomics

AI has revolutionized single-cell transcriptomics in lung cancer, providing deep insights into cellular heterogeneity, the tumor microenvironment, cancer stem cells, and therapeutic responses and resistance mechanisms. Single-cell transcriptomics, a high-throughput sequencing technology, enables detailed gene expression analysis at the single-cell level, essential for understanding lung cancer pathogenesis and immune evasion. AI algorithms, such as clustering techniques, identify and classify different cell populations within tumors, unveiling intratumoral heterogeneity (83). ML uncovers critical genes associated with specific cell types, delineating unique cellular characteristics (84).

AI reconstructs cellular development trajectories, shedding light on transitions from normal to malignant cells. In the tumor microenvironment, AI analyzes ligand-receptor interactions and integrates single-cell RNA-seq with spatial transcriptomics to map the spatial distributions of cells (85). It also enhances the identification of immune cell populations, deepening the understanding of immune evasion. In cancer stem cell research, AI identifies specific markers and drug resistance mechanisms, unveiling potential therapeutic targets (86). AI predicts therapeutic responses by analyzing pretreatment and posttreatment transcriptomics data, aiding personalized treatment strategies and showcasing the potential of AI potential in precision medicine for lung cancer (86).

#### 2.2.5 AI in spatiotemporal transcriptomics of lung cancer

In the realm of spatial-temporal transcriptomics, AI has become indispensable for dissecting the intricate landscape of lung cancer. This emerging technology scrutinizes cellular transcriptional activity across different spatial and temporal contexts, offering insights into cellular interactions, migration patterns, and the tumor microenvironment. AI-based algorithms, including ML, DL, and image processing, handle the massive datasets generated, facilitating the integration of data across various time points and spatial locations (87, 88). Through AI-enhanced image analysis, such as cell segmentation and microenvironmental parsing, DL methods like CNNs can automatically identify and classify cell types and structures within complex images. Pattern recognition capabilities of AI further enable the identification of dynamic gene expression patterns and interactions among cellular populations, essential for predicting disease progression and therapeutic responses. Dimensionality reduction and visualization techniques, optimized by AI, such as Principal Component Analysis (PCA), t-Distributed Stochastic Neighbor Embedding (t-SNE), and Uniform Manifold Approximation and Projection (UMAP), provide intuitive interpretations of high-dimensional data, revealing the spatial and temporal distribution of different cell types and gene expressions. Moreover, AI can elucidate gene-environment interactions, pinpointing how spatial and temporal variations impact lung cancer development. This approach also aids in predicting drug responses, paving the way for personalized therapies by associating specific gene expression profiles with drug sensitivity or resistance. The integration of AI in spatial-temporal transcriptomics not only enhances the accuracy and efficiency of data analysis but also empowers researchers to unravel the complex biological processes underlying lung cancer, driving the advancement of novel therapeutic strategies.

#### 2.2.6 AI in transcription factor regulatory networks in lung cancer

AI has shown immense potential in enhancing the understanding and treatment of lung cancer by focusing on transcription factor regulatory networks. These networks, which involve complex interactions between transcription factors and their target genes, are critical in lung cancer development and progression. AI excels in managing and integrating vast datasets, such as gene expression and clinical data, revealing hidden patterns and associations. Techniques like genomics and transcriptomics allow AI to analyze high-throughput sequencing data, identifying crucial transcription factors and target genes. Additionally, AI algorithms, including ML and DL, facilitate the construction and inference of intricate regulatory networks (89).ML algorithms play a pivotal role in selecting key regulatory factors from extensive datasets, highlighting potential therapeutic targets. AI also contributes to the discovery of novel biomarkers for early diagnosis and prognosis of lung cancer. In drug discovery and repurposing, AI-driven virtual screening identifies compounds that disrupt critical regulatory networks, offering new therapeutic avenues. Moreover, AI supports personalized medicine by integrating genomic, regulatory network, and clinical data to formulate individualized treatment plans and predict patient outcomes. By modeling and simulating regulatory networks, AI aids in uncovering underlying biological mechanisms, thereby advancing our comprehension of lung cancer. The promising applications of AI in this domain are expected to drive innovative solutions and propel lung cancer research and treatment forward.

### 2.3 AI in proteomics of lung cancer

Proteomics, the large-scale study of proteins, generates massive datasets that require sophisticated tools for efficient and accurate analysis. AI excels in this aspect, offering rapid data processing and analysis capabilities. ML algorithms are adept at recognizing complex patterns within these datasets, identifying key protein expression profiles and biomarkers associated with lung cancer. This enables researchers to discern important biological insights that traditional methods might overlook.

In the realm of feature selection and classification, AI plays a pivotal role. It can efficiently identify which protein features are most distinctive between lung cancer patients and healthy individuals, thereby enhancing diagnostic precision (90). AI-based classification models, such as decision trees, random forests, and SVM, can predict the presence of lung cancer in samples with high accuracy (91). Moreover, the application of ML algorithms in biomarker discovery has been transformative. These algorithms analyze proteomics data to identify potential biomarkers for early diagnosis, prognosis prediction, and monitoring therapeutic efficacy (92). DL models further augment this process by extracting high-dimensional features from complex data, revealing insights that might be missed by conventional approaches.

AI also contributes to the construction of protein interaction networks, which are crucial for understanding the molecular mechanisms of lung cancer (93). By performing network analyses, AI can identify key nodes and pathways, thereby highlighting critical points of intervention and potential drug targets. Additionally, AI tools can predict protein-protein interactions that are pivotal in lung cancer progression, providing deeper biological understanding and guiding the development of targeted therapies.

Lastly, the automation and optimization of experimental design through AI significantly enhance research efficiency and cost-effectiveness. AI can design and optimize experiments, reducing the number of required trials and associated costs while increasing accuracy. By simulating and modeling experimental outcomes, researchers can expedite their studies, accelerating the overall research process. Overall, the integration of AI in lung cancer proteomics has exponentially expanded the depth and breadth of biomedical research, facilitating the discovery of effective diagnostic and therapeutic methods with unprecedented speed and precision.

### 2.4 AI in lung cancer metabolomics

AI has been prominently utilized in the field of metabolomics for lung cancer, significantly advancing both research methods and clinical practices. Lung cancer metabolomics, the study and analysis of metabolic changes within patients, involves handling vast and complex datasets generated by high-throughput technologies such as mass spectrometry and nuclear magnetic resonance spectroscopy. These initial datasets often contain significant noise and complex structures, necessitating robust processing techniques. AI plays a critical role in this phase by automating data cleaning and denoising, ensuring high-quality results by removing outliers and reducing noise. Furthermore, AI employs ML and DL algorithms for feature extraction, discerning vital metabolic features pertinent to lung cancer diagnosis and research (94, 95). It integrates data from diverse sources like blood, tissue, and urine samples, creating a comprehensive database. For instance, using autoencoders in ML, researchers can capture nonlinear variations and distinguish significant metabolic features between cancer patients and healthy controls, enhancing data processing efficiency and accuracy (95, 96).

The contribution of AI extends to the discovery of biomarkers critical for early diagnosis and treatment monitoring (96). Through pattern recognition techniques, such as supervised or unsupervised learning algorithms including neural networks, SVM, and random forests, AI identifies metabolites significantly associated with lung cancer, pinpointing potential new diagnostic biomarkers. Additionally, AI bolsters disease classification and personalized diagnosis by utilizing DL models on metabolomics data to distinguish lung cancer subtypes and even precancerous lesions automatically (97). These models, like convolutional neural networks, enable high-accuracy classification supporting the development of personalized treatment strategies based on individual metabolic data. Moreover, AI aids in prognostic prediction, leveraging metabolomics data to forecast disease progression and patient outcomes (98). By integrating AI with survival analysis models such as Cox regression, researchers can identify key prognostic factors, predict survival time of patients and recurrence risk, aiding in tailored therapeutic decision-making. Furthermore, in drug development and repurposing, AI utilizes metabolomics data to identify target enzymes and proteins, predict drug efficacy, and explore existing new potential uses for drugs in lung cancer treatment, thereby expediting drug development processes.

### 2.5 AI in lung cancer immuno-oncology

“Lung cancer immunomics” is an emerging term that combines immunology and omics research to delve into the immune mechanisms involved in lung cancer and their relationship with genomic characteristics. Our review primarily focuses on this topic, emphasizing the application of AI in studying tumor immune evasion mechanisms, immune-related gene mutations, tumor mutation burden (TMB), neoantigen discovery, immune microenvironment analysis, and the personalization of immunotherapy.

#### 2.5.1 AI in immune evasion of lung cancer

The application of AI in studying the immune escape mechanisms in lung cancer has yielded significant advancements, bringing a more nuanced understanding of the complex tumor-immune interactions. One crucial area of application is the immunophenotyping analysis, where DL and ML techniques are utilized to analyze Fluorescence-activated Cell Sorting (FACS) data (99). This allows for the precise identification and classification of various immune cell subtypes, elucidating their roles in immune escape (100). Another pivotal use of AI lies in the analysis of T-cell receptor (TCR) and B-cell receptor (BCR) repertoires. ML models can decode the complex clonal dynamics associated with immune escape by analyzing the rearrangement sequences of TCRs and BCRs (101). Additionally, single-cell immunogenomics, supported by AI algorithms, has enabled a granular investigation of the functional states and interactions of heterogeneous immune cells within the Tumor Microenvironment (TME) (102). AI techniques also contribute to a comprehensive analysis of immune escape-related genes and pathways, identifying key drivers and mechanisms of immune evasion. In the realm of therapeutic applications, ML models predict patient responses to immunotherapies, such as Programmed Death-1/Programmed Death-Ligand 1 (PD-1/PD-L1) inhibitors, thereby aiding in the personalization of treatment strategies (101). Moreover, through the simulation and modeling of the tumor immune microenvironment, AI provides insights into how cancer cells exploit these alterations to evade immune surveillance, identifying novel therapeutic targets (102). Collectively, AI not only enhances our understanding of lung cancer immune escape mechanisms but also plays a crucial role in the development of more effective immunotherapies (101).

#### 2.5.2 AI in immune-related gene mutations of lung cancer

The ability of AI to process vast amounts of genomic data has proven indispensable in identifying mutations pertinent to the immune response, such as those in the PD-L1, Cytotoxic T-Lymphocyte Antigen 4 (CTLA-4), or Human Leukocyte Antigen (HLA) genes (103). By decoding these genetic variations, AI aids in elucidating the interactions between tumors and the immune system, thereby facilitating the design of personalized treatment plans.

Furthermore, AI significantly enhances the prediction of responses to immunotherapy. By scrutinizing immune-related genetic information, AI models can forecast their potential reactions to immunotherapeutic agents like PD-1/PD-L1 inhibitors (99). This predictive capability enables clinicians to tailor immunotherapy regimens more accurately, optimizing therapeutic efficacy and reducing the likelihood of adverse reactions. The integration of AI into the analysis of immune-related gene mutations thus represents a transformative progression in lung cancer treatment, providing deeper insights and fostering more precise interventions (104).

As our understanding of the complex interplay between the immune system and cancer deepens, AI-driven approaches are poised to become even more central in identifying vital genetic mutations and tailoring immunotherapies. The implications for personalized medicine are profound, underscoring the necessity for continued research and development in this promising intersection of AI and lung cancer immunogenomics.

#### 2.5.3 AI in TMB of lung cancer

AI has emerged as a transformative tool in the estimation and prediction of TMB in lung cancer. Utilizing comprehensive whole-genome sequencing data or specific gene panel data, AI, powered by advanced DL and ML algorithms, can meticulously analyze extensive genomic information. This enables the precise estimation of TMB, which is crucial for understanding tumor profile (105). Moreover, TMB is a significant biomarker for determining the efficacy of immunotherapies, such as PD-1/PD-L1 inhibitors. High TMB levels often correlate with better responses to these immuno-oncology agents. By leveraging AI to integrate TMB data with other clinical parameters, healthcare professionals can develop more personalized treatment plans, thereby enhancing therapeutic outcomes. Additionally, AI models that combine TMB with other biomarkers can provide valuable prognostic insights, aiding in the prediction of lung cancer patients’ outcomes. This prognostic capability is vital for shaping long-term treatment strategies and follow-up plans. The continuous advancements in AI technologies herald a promising future for its application in the precise and personalized management of lung cancer, particularly through the lens of TMB analysis.

#### 2.5.4 AI in the discovery of neoantigens in lung cancer immunotherapy

AI models excel in predicting the likelihood of mutant proteins being processed into peptide fragments recognizable as neoantigens by the immune system, with tools such as DeepNovo or PepFormer aiding in this endeavor (106, 107). Additionally, AI algorithms critically evaluate the stability of these peptides within biological environments, ensuring their viability as neoantigens. The binding affinity of mutated peptides to Major Histocompatibility Complex (MHC) molecules is a crucial determinant of neoantigen effectiveness, with machine learning models like NetMHCpan and MHCflurry being instrumental in this prediction (108, 109). AI also models the complex process of neoantigen generation and MHC presentation, filtering out the most promising candidates. This AI-driven approach not only enhances the accuracy of neoantigen identification but also accelerates the development of personalized cancer immunotherapies.

#### 2.5.5 AI in immune microenvironment of lung cancer

AI algorithms have demonstrated remarkable efficacy in single-cell RNA sequencing analysis, enabling the identification and classification of various immune cell types within the TME, such as T cells, B cells, and natural killer (NK) cells (101). ML techniques further facilitate lineage tracing, revealing the developmental and differentiation pathways of immune cells in the TME (102). Additionally, AI aids in functional gene expression analysis, evaluating immune cell states such as activation, inhibition, and exhaustion, and constructing interaction networks between immune cells and tumor or stromal cells, elucidating their functional roles within the TME. AI technologies also excel at characterizing immunosuppressive microenvironments by identifying factors like Tregs and Myeloid-Derived Suppressor Cells (MDSCs), and their impact on immune responses. Dynamic monitoring of the TME is achieved through AI-assisted liquid biopsy technologies, enabling real-time surveillance of ctDNA and immune cell states, thus assessing immunotherapy efficacy. Furthermore, using ML for spatial analysis of TME can predict the efficacy of immunotherapy in SCLC patients (110). The crucial role of AI in lung cancer immunology research promises even greater advancements in the field, heralding innovative and breakthrough diagnostics and therapies.

### 2.6 AI in lung cancer microbiomics

AI technologies, notably DL and ML algorithms, have become pivotal in several aspects of microbiome research. Through AI-driven approaches, researchers can accurately identify and classify microbial species, as well as assess their relative abundances within the lung microbiome (111). This capability is invaluable, given the complexity and sheer volume of data generated by high-throughput sequencing techniques.

One key area of AI application is the prediction of microbial functions and metabolic pathways. By analyzing microbial genomes using AI models, researchers can infer potential functions and metabolic activities of various microbes, thereby elucidating their roles in the pathogenesis and progression of lung cancer (111). This functional prediction is crucial for understanding how specific microbes may contribute to cancer development, providing insights that could lead to new therapeutic strategies.

Pattern recognition and feature extraction are another vital area where AI proves indispensable. ML algorithms are adept at extracting significant features from complex microbiome data sets (112). These features can then be utilized to explore the associations between certain microbial species or communities and lung cancer. This process aids in the identification of microbiome markers that could be correlated with disease presence, progression, or even patient outcomes.

In addition, AI enhances the ability to perform comprehensive association analyses by integrating clinical characteristics with microbiome data to identify microbial biomarkers related to lung cancer. This integration facilitates the identification of potential microbial indicators that could be used in diagnostic assays or as therapeutic targets. Furthermore, AI algorithms are employed to build and validate predictive models for lung cancer risk assessment, enhancing early screening and diagnostic accuracy by incorporating both microbiome and clinical features (113). Such risk assessment models are crucial for early intervention and improving patient prognosis.

Prognostic models based on microbiome data powered by AI can also predict patient outcomes, including survival time and response to treatment (113). This predictive capability is essential for personalized medicine, as it allows for tailored therapeutic approaches based on unique microbiome profile. Additionally, AI aids in drug discovery and development, where it can be used to screen for potential anti-cancer or health-promoting microbial metabolites. By identifying these promising compounds, AI can contribute to the development of new therapeutics for lung cancer treatment.

In summary, the applications of AI in lung cancer microbiome research have significantly accelerated the research process, providing powerful tools for precise diagnosis, personalized treatment, and the development of new drugs. The integration of AI with microbiome research holds immense potential to advance our understanding of lung cancer and improve patient outcomes.

### 2.7 AI in radiomics of lung cancer

Radiomics, as a computational technique, focuses on extracting a vast array of features from medical images, which include parameters such as shape, texture, intensity, and wavelet attributes. Detailed analysis of these features provides comprehensive information about lung tumors, aiding in diagnosis, prognostic prediction, and the assessment of therapeutic responses. AI’s application in lung cancer radiomics is extensive, significantly enhancing the automation and accuracy of image analysis.

One of the primary applications of AI in lung cancer radiomics is automated image segmentation. Utilizing DL algorithms, particularly CNNs, AI can precisely segment tumor regions within lung images, thereby minimizing human error and reducing time consumption (16). Architectures such as U-Net and Mask R-CNN are extensively applied in medical image segmentation, demonstrating high efficacy in delineating tumor boundaries from surrounding tissues (114, 115). In terms of feature extraction and selection, ML algorithms play a pivotal role. These algorithms automatically extract high-dimensional imaging features, such as shape, texture, and wavelet characteristics, and subsequently select features with the most significant diagnostic and prognostic value (116). Techniques like Random Forest, SVM, and Principal Component Analysis (PCA) are commonly employed for feature selection and dimensionality reduction, enabling the creation of robust predictive models.AI-driven diagnostic support systems, such as computer-aided diagnosis (CAD), enhance early detection accuracy by identifying abnormalities in CT scans and highlighting regions of interest for clinicians. Notable systems like IBM Watson Health and Google-owned DeepMind have developed advanced solutions that support early lung cancer diagnosis, underscoring the potential of AI in clinical practice.

Prognostic prediction models, powered by DL and other ML techniques, analyze radiomic data to forecast patient outcomes, including survival rates, disease progression risks, and treatment responses (117). For instance, by combining survival analysis models with imaging features, personalized prognostic predictions can be made, aiding in informed clinical decision-making (118).

AI also significantly contributes to personalized treatment planning and evaluation. By analyzing radiomic features, AI algorithms recommend tailored treatment plans, encompassing the optimal combination of immunotherapy, surgery, and radiotherapy (119–121).

To handle the extensive volume of radiomic data, AI leverages cloud computing and distributed computing technologies. Platforms such as Amazon Web Services (AWS) and Google Cloud provide scalable solutions for efficiently processing and analyzing large-scale medical imaging datasets, facilitating advanced research and clinical applications.

Furthermore, AI-enhanced radiomics is instrumental in predicting lymph node metastasis in early-stage NSCLC and improving the accuracy and efficiency of lung nodule detection and diagnosis during screenings. Studies combining AI with Positron Emission Tomography/Computed Tomography (PET/CT) imaging have demonstrated significant advancements in detecting and evaluating NSCLC, particularly in identifying occult lymph node metastasis and predicting responses to immunotherapy based on radiomic features (122).

### 2.8 Leveraging AI for pathomics in lung cancer

AI represents a transformative technology in the domain of pathological histology of lung cancer, offering substantial potential to revolutionize diagnostic accuracy and efficiency, optimization of treatment plans, and support for personalized medicine. Pathological histology, referring to the microscopic study of lung cancer tissues, is integral to the precise diagnosis, categorization, staging, and formulation of targeted treatment strategies. By leveraging AI in this field, clinicians and pathologists can significantly enhance the accuracy and speed of diagnostic processes. DL technologies, particularly CNNs, are adept at automating the analysis of pathological tissue section images (123). These AI algorithms can identify and quantify the presence, type, density, and distribution of cancer cells, thus aiding pathologists in making rapid and accurate diagnoses.

Furthermore, AI-driven image classification systems classify pathological images into distinct lung cancer types, thereby facilitating more precise diagnostic outcomes (124, 125). In terms of feature extraction and quantification, AI systems can harvest a plethora of data from pathological images, including cell morphology, arrangement patterns, and staining intensity, which are critical for assessing cancer severity and progression (126). The role of AI extends beyond diagnosis by also predicting patient prognosis based on a combination of pathological images and clinical data, such as survival rates and recurrence risks (127). This predictive capability is essential for formulating personalized treatment plans tailored to individual patient profiles.

Additionally, the ability of AI to predict treatment response by analyzing genomic data alongside pathological images enables the selection of the most effective treatment modalities, such as chemotherapy, targeted therapy, or immunotherapy, suited to unique characteristics (128). AI also excels in data integration and information management, combining data from diverse sources, including genomic data, electronic health records (EHRs), and pathological images, to provide comprehensive patient information that supports critical decision-making processes. By automating repetitive workflows, such as digitizing tissue sections, pre-processing images, and performing preliminary analyses, AI enhances the efficiency of pathology laboratories.

Real-time diagnostic assistance during microscopic examinations further exemplifies the capability of AI, offering diagnostic suggestions that help pathologists detect subtle abnormalities (129). The AI systems’ ability to continuously learn and improve from new data ensures sustained accuracy in diagnostic results. Moreover, AI platforms facilitate cross-institutional collaboration and knowledge sharing by enabling the sharing of data and models, fostering innovation and collective expertise in pathological research.

Several studies illustrate the practical application of AI in pathological histology of lung cancer. For instance, researchers at the University of Washington utilized ML algorithms to predict brain metastasis in early-stage non-small cell lung cancer using 118 lung biopsy samples (130). At the University of Texas Southwestern Medical Center, the combination of AI with traditional pathology expedited the formulation of treatment plans (131). Researchers at New York University Langone Health demonstrated that AI tools capable of autonomous learning could analyze lung tissues to predict cancer recurrence, aiding in the prognostication and severity assessment of lung cancer (132).

An article in “Nature” discusses PathChat, a multimodal generative AI assistant specifically developed for human pathologists (20). By utilizing visual language models, PathChat effectively processes and integrates various data modalities. This innovative approach significantly enhances diagnostic accuracy and efficiency, offering robust support to pathologists in their professional analyses. The future applications of AI in lung cancer pathological histology are vast, promising substantial improvements in diagnostic precision and efficiency while advancing personalized medicine and pathological research.

## 3 The new era of precision oncology: AI-driven revolution in lung cancer care and future prospects

The field of precision medicine in lung cancer is undergoing a paradigm shift, largely driven by advances in AI. Precision medicine refers to the approach of tailoring medical treatment to the individual characteristics of each patient, such as genetic profile, tumor molecular characteristics, and other biomarkers. By aligning treatment options with the unique biological context of each patient, precision medicine aims to enhance therapeutic efficacy while minimizing unnecessary adverse effects. AI holds considerable promise in the future of lung cancer precision medicine, including early diagnosis, personalized treatment regimens, treatment monitoring, and new drug development.

For early diagnosis, the potential of AI potential in image recognition is monumental. By applying AI and image recognition technologies to vast collections of CT scans, AI can automatically identify early-stage lung nodules and minute pathological changes, thereby facilitating early diagnosis and increasing the chances of successful treatment (133). This capability is being seamlessly integrated into clinical workflows, enhancing the efficiency and accuracy of radiologists’ work. For instance, AI-powered computer-aided detection (CAD) systems are now being employed in many hospitals to assist radiologists in detecting and characterizing lung nodules on CT scans. These systems can rapidly analyze hundreds of images, flagging suspicious areas for further review by radiologists. This not only speeds up the diagnostic process but also improves the detection rate of small nodules that might be overlooked by human eyes alone. Moreover, AI algorithms are being developed to differentiate between benign and malignant nodules, potentially reducing unnecessary biopsies and follow-up scans. For example, a deep learning model developed by researchers at Google Health and Northwestern University demonstrated the ability to detect lung cancer from CT scans with a performance on par with or better than radiologists (134). Such tools are gradually being incorporated into clinical practice, serving as a “second reader” to support radiologists’ decision-making processes. AI’s role in early detection extends beyond imaging. Complementarily, AI’s ability to analyze ctDNA from liquid biopsies could detect genetic mutations associated with lung cancer at an early stage (135). This non-invasive approach is particularly promising for screening high-risk populations and monitoring disease recurrence. AI algorithms can analyze complex patterns in ctDNA data, potentially identifying cancer-specific signatures before traditional diagnostic methods can detect the disease (136). In pathology, AI’s automated analysis of histopathological slides can swiftly and accurately identify cancerous regions, thereby improving diagnostic precision and efficiency. These AI systems are being integrated into digital pathology workflows, assisting pathologists in quantifying biomarkers, grading tumors, and identifying specific histological patterns associated with different lung cancer subtypes (126).Importantly, these AI applications are not limited to NSCLC but are also being developed and refined for SCLC detection. Given the aggressive nature of SCLC and the critical importance of early detection, AI tools are being tailored to identify the unique radiological and pathological features of SCLC, potentially leading to earlier diagnosis and improved outcomes for this challenging subtype of lung cancer (137).

In the realm of personalized treatment plans, AI promises to revolutionize gene analysis and targeted therapy. AI systems, leveraging DL and extensive datasets, will be able to process and analyze large volumes of genetic data to pinpoint mutations and biomarkers associated with lung cancer (138, 139). By predicting the response of specific genetic profiles to targeted therapies, AI can help devise highly customized treatment protocols (140). Additionally, AI can integrate multidisciplinary data, encompassing genetic information, imaging data, clinical records, and lifestyle habits, to holistically evaluate patient health (133). This multimodal approach will lead to more precise and effective treatment plans, improving therapeutic outcomes and reducing side effects. Furthermore, adaptive AI systems will dynamically adjust treatment strategies based on real-time data, such as changes in biomarker concentrations or imaging results, thus refining the precision and personalization of lung cancer therapies.

For cases of advanced-stage cancer, AI is increasingly aiding oncologists in creating personalized treatment plans, leveraging predictive analytics to optimize chemotherapy, targeted therapies, and immunotherapies. For instance, AI algorithms are being developed to predict the efficacy of tyrosine kinase inhibitors (TKIs) in patients with specific EGFR mutations, allowing for more precise treatment selection (141). Similarly, AI models are being used to predict response to ALK inhibitors in patients with ALK-positive NSCLC, potentially guiding treatment decisions and improving outcomes (142).Additionally, AI can integrate multidisciplinary data, encompassing genetic information, imaging data, clinical records, and lifestyle habits, to holistically evaluate patient health (143). This multimodal approach leads to more precise and effective treatment plans, improving therapeutic outcomes and reducing side effects. For example, AI models that incorporate radiomics features from CT scans, along with clinical and genetic data, have shown promise in predicting response to immunotherapy in NSCLC patients (144).Furthermore, adaptive AI systems can dynamically adjust treatment strategies based on real-time data, such as changes in biomarker concentrations or imaging results, thus refining the precision and personalization of lung cancer therapies. This is particularly valuable in managing treatment-related toxicities and adapting dosages to maximize efficacy while minimizing side effects. In the rapidly evolving sphere of oncology, particularly within the realm of immunotherapy for lung cancer patients, the integration of AI has introduced significant advancements in therapeutic precision and personalization. This burgeoning field is underscored by recent research that showcases the development of cutting-edge AI and machine learning models, which are meticulously designed to predict patient responses to immunotherapy (145, 146). These models extend their utility by forecasting progression-free survival and overall survival rates, specifically tailored for individuals battling NSCLC (147). Such AI-driven tools leverage standard clinical data to evaluate the efficacy of immune checkpoint inhibitors, thus equipping clinicians with vital insights that inform the selection of optimal treatment regimens. Consequently, these technological innovations are revolutionizing the landscape of personalized medicine, granting healthcare providers the capability to discern which patients are predisposed to benefit most from specific immunotherapy treatments.

In the context of treatment monitoring, AI’s ability to conduct real-time data surveillance is nothing short of transformative. By enabling the continuous monitoring of vital physiological metrics, genetic profiles, and treatment responses through sophisticated smart devices and sensors, AI facilitates timely modifications to treatment plans that align with evolving data patterns, thereby maximizing therapeutic efficacy. For instance, AI algorithms can analyze serial CT scans to assess tumor response to treatment, potentially detecting subtle changes that might indicate the need for treatment modification before clinical symptoms appear (148). Similarly, AI models can monitor changes in circulating tumor DNA levels during treatment, providing early indications of treatment response or resistance (64).Furthermore, predictive models empowered by AI serve a pivotal role in not only forecasting treatment outcomes and patient prognoses but also in crafting individualized predictions regarding disease trajectories (110, 149, 150). These insights are invaluable in helping clinicians devise comprehensive, long-term management strategies. For example, AI models that integrate clinical, pathological, and genomic data have shown promise in predicting long-term survival in NSCLC patients, potentially guiding decisions about treatment intensity and follow-up protocols. The importance of AI is further emphasized by its capacity to predict and signal potential complications through real-time analysis of physiological data and clinical records, which is crucial in preempting adverse medical events and guiding proactive interventions, ultimately mitigating the risks associated with immunotherapy treatments.

In the realm of drug development for lung cancer, significant strides have been made through the integration of AI. AI platforms have revolutionized the pharmaceutical industry by enhancing the speed and reducing the costs of drug discovery processes (151). AI are being used to streamline various stages, from identifying druggable targets to optimizing lead compounds (152–154). AI’s ability to rapidly analyze biological datasets is exemplified by projects like those at Lawrence Livermore National Laboratory and BridgeBio, which have advanced to clinical trials for medications targeting genetic mutations in cancer. The application of AI facilitates virtual drug screening, optimizes therapeutic molecule development, and enhances clinical trial design, thereby shortening trial durations and minimizing costs (155). Additionally, AI is pivotal in personalized medicine, creating tailored treatments based on genetic profiles, thus maximizing efficacy and minimizing side effects compared to conventional approaches.

The future applications of AI in lung cancer precision medicine are vast and multifaceted. AI is playing an increasingly pivotal role in early detection, personalized treatment planning, treatment monitoring, and new drug development. By harnessing the power of genetic, clinical, and imaging data, AI can deliver highly accurate and effective medical solutions, significantly improving patient survival rates and quality of life. As technology advances, the integration of AI into lung cancer precision medicine will continue to expand and deepen, heralding a new era of personalized healthcare. The seamless incorporation of AI tools into clinical workflows is enhancing the ability to detect both NSCLC and SCLC at earlier stages, while also providing oncologists with powerful decision support systems for crafting personalized treatment plans for advanced-stage patients. However, it’s important to note that while AI holds great promise, its implementation in clinical practice should be done cautiously and ethically. Rigorous validation studies, regulatory approvals, and ongoing monitoring of AI systems in real-world settings are crucial to ensure their safety and efficacy. Additionally, efforts should be made to address potential biases in AI algorithms and to ensure equitable access to AI-enhanced healthcare across diverse populations. As we move forward, the synergy between human expertise and AI capabilities will likely define the future of lung cancer care, offering hope for improved outcomes and quality of life for patients worldwide.

## 4 Technical challenges and solutions

AI holds considerable promise for advancing lung cancer diagnosis, treatment, and prognosis. Nonetheless, realizing this potential requires addressing several key challenges related to data quality and quantity, model interpretability, statistical validation, and ethical and privacy considerations.

Initially, addressing data quality and quantity is crucial. Medical datasets often contain noise, missing values, and errors due to human input, all of which can adversely affect AI model performance. Moreover, the development of large-scale, high-quality annotated datasets is fraught with difficulties, limiting the robustness and generalizability of AI models. Data heterogeneity further complicates this issue, as variations across healthcare institutions or imaging devices often lead to a lack of standardization. Additionally, integrating diverse data types, such as imaging, genomic, and clinical records, calls for sophisticated multimodal analysis.

To confront these challenges, several strategies are recommended. Implementing rigorous data cleaning and standardization protocols can significantly mitigate noise and rectify errors, thereby improving data quality. Enhancing data volume through multi-center collaborations and secure data-sharing platforms is essential, all while maintaining strict data privacy and security. Furthermore, employing data augmentation techniques, including image rotation, translation, and scaling, can bolster training sample sizes. The creation of unified data formats and processing standards is crucial for the standardization and integration of multimodal data, thereby maximizing the utility of available information.

Equally important is the challenge of model interpretability, particularly with DL models like CNNs. While CNNs excel in image diagnostics, their “black box” nature makes understanding their decision-making processes difficult, thus impeding trust in their predictions. To enhance interpretability, several approaches are suggested. Employing Explainable AI (XAI) techniques, such as Gradient-weighted Class Activation Mapping (Grad-CAM), helps produce heat maps that visualize decision-making focal areas. Additionally, incorporating attention mechanisms into CNNs analyzing CT scans can highlight suspicious areas, providing visual cues of the decision process to clinicians. The use of Local Interpretable Model-agnostic Explanations (LIME) can further clarify individual predictions, enhancing clinicians’ comprehension of data feature contributions. A notable addition to these interpretability methods is the SHapley Additive exPlanations (SHAP) approach. SHAP, based on game theory concepts, offers a unified framework for interpreting predictions. It provides both global and local explanations of model behavior, which is particularly valuable in medical imaging where understanding the importance of different image regions in model decisions is crucial. SHAP’s model-agnostic nature and strong theoretical foundation make it especially suitable for complex deep learning models used in lung cancer detection, potentially increasing clinicians’ trust in AI-assisted diagnoses.

In parallel, rigorous statistical validation is needed to ensure the reliability and generalizability of AI models in lung cancer research. Applying stratified k-fold cross-validation ensures that cancer stages or subtypes are consistently represented across folds, yielding robust model performance estimates. External validation on independent datasets is critical for assessing model performance on unobserved data from various institutions, unveiling potential biases or limitations. Comprehensive performance metrics should also be evaluated, including Area Under the Curve of Receiver Operating Characteristic (AUC-ROC), precision-recall curves, and F1 scores, alongside traditional metrics. Calibration plots and decision curve analysis can effectively gauge the clinical utility of these models across different threshold probabilities.

Moreover, ethical and privacy considerations are foundational to the application of AI in healthcare. The use of patient data raises complex ethical issues, necessitating robust safeguards for privacy protection and adherence to ethical guidelines. Transitioning from research to clinical practice further underscores the need for models capable of operating reliably across diverse clinical environments and conditions.

In summary, systematically addressing these challenges through strategic measures can markedly enhance the applicability and effectiveness of AI in lung cancer research and clinical settings. Enhancing model interpretability and ensuring thorough statistical validation are key to building clinician trust and encouraging clinical integration. Developing comprehensive interpretability frameworks and validation protocols will enable healthcare professionals to confidently rely on AI diagnostic results, thereby improving diagnostic accuracy and treatment efficacy for patients.

## 5 Conclusion

The integration of AI into lung cancer research and treatment has already demonstrated substantial advancements, enhancing diagnostic accuracy, treatment planning, and patient outcome prediction. AI’s application across various -omics fields, radiomics, and pathology has provided unprecedented insights into the molecular mechanisms of lung cancer, facilitating early detection and personalized treatment strategies. Despite challenges such as data quality, interpretability, and ethical considerations, ongoing technological advancements and strategic solutions promise to overcome these hurdles. The future of AI in lung cancer is poised for transformative impact, with potential to revolutionize precision medicine, accelerate drug discovery, and improve patient care. As AI continues to evolve, its role in lung cancer management will deepen, offering innovative solutions and contributing to a more comprehensive understanding of the disease, ultimately leading to better patient outcomes and enhanced quality of life.

## Glossary

AI: Artificial Intelligence
NSCLC: Non-small cell lung cancer
SCLC: Small cell lung cancer
EGFR: Epidermal Growth Factor Receptor
ALK: Anaplastic Lymphoma Kinase
ML: Machine learning
DL: Deep Learning
NLP: Natural Language Processing
DNNs: Deep neural networks
CT: Computed Tomography
CNNs: Convolutional Neural Networks
RNNs: Recurrent Neural Networks
KRAS: Kirsten Rat Sarcoma virus
TCGA: The Cancer Genome Atlas
GDC: Genomic Data Commons
CNV: Copy number variation
GEO: Gene Expression Omnibus
ncRNA: non-coding RNA
miRNAs: microRNAs
lncRNAs: long non-coding RNAs
ctDNA: circulating tumor DNA
dPCR: Digital Polymerase Chain Reaction
NGS: Next-generation sequencing
RNA-Seq: RNA Sequencing
mRNA: messenger RNA
SVMs: Support vector machines
circRNAs: circular RNAs
m6A: N6-methyladenosine
PCA: Principal Component Analysis
t-SNE: t-Distributed Stochastic Neighbor Embedding
UMAP: Uniform Manifold Approximation and Projection
TMB: Tumor mutation burden
FACS: Fluorescence-activated Cell Sorting
TCR: T-cell receptor
BCR: B-cell receptor
TME: Tumor Microenvironment
PD-1: Programmed Death-1
PD-L1: Programmed Death-Ligand 1
CTLA-4: Cytotoxic T-Lymphocyte Antigen 4
HLA: Human Leukocyte Antigen
MHC: Major Histocompatibility Complex
NK: Natural killer
MDSCs: Myeloid-Derived Suppressor Cells
PCA: Principal Component Analysis
CAD: computer-aided diagnosis
TKIs: Tyrosine kinase inhibitors
AWS: Amazon Web Services
PET/CT: Positron Emission Tomography/Computed Tomography
EHRs: Electronic health records
XAI: Explainable Artificial Intelligence
HIPAA: Health Insurance Portability and Accountability Act
GDPR: General Data Protection Regulation
Grad-CAM: Gradient-weighted Class Activation Mapping
LIME: Local Interpretable Model-agnostic Explanations
SHAP: SHapley Additive exPlanations
AUC-ROC: Area Under the Curve of Receiver Operating Characteristic
